# Protocol for iGrow (Infant Growth and Development Study): biopsychosocial predictors of childhood obesity risk at 2 years

**DOI:** 10.1186/s12889-020-10003-0

**Published:** 2020-12-14

**Authors:** Esther M. Leerkes, Cheryl Buehler, Susan D. Calkins, Lenka H. Shriver, Laurie Wideman

**Affiliations:** 1 UNC Greensboro, Department of Human Development and Family Studies, Greensboro, NC 27402-6170 USA; 2 UNC Greensboro, Office of Research and Engagement, Greensboro, NC 27402-6170 USA; 3 UNC Greensboro, Department of Nutrition, Greensboro, NC 27402-6170 USA; 4 UNC Greensboro, Department of Kinesiology, Greensboro, NC 27402-6170 USA

**Keywords:** Childhood obesity, Infant weight gain, Feeding practices, Parenting, Child self-regulation, Hormones, Inflammatory makers, Prenatal, Postnatal, Prospective longitudinal study

## Abstract

**Background:**

Childhood obesity remains a significant public health problem. To date, most research on the causes and correlates of obesity has focused on a small number of direct predictors of obesity rather than testing complex models that address the multifactorial nature of the origins of obesity in early development. We describe the rationale and methods of iGrow (Infant Growth and Development Study) which will test multiple pathways by which (a) prenatal maternal psychobiological risk predicts infant weight gain over the first 6 months of life, and (b) this early weight gain confers risk for obesity at age 2. Infant hormonal and psychobiological risk are proposed mediators from prenatal risk to early weight gain, though these are moderated by early maternal sensitivity and obesogenic feeding practices. In addition, higher maternal sensitivity and lower obesogenic feeding practices are proposed predictors of adaptive child self-regulation in the second year of life, and all three are proposed to buffer/reduce the association between high early infant weight gain and obesity risk at age 2.

**Methods:**

iGrow is a prospective, longitudinal community-based study of 300 diverse mothers and infants to be followed across 5 data waves from pregnancy until children are age 2. Key measures include (a) maternal reports of demographics, stress, well-being, feeding practices and child characteristics and health; (b) direct observation of maternal and infant behavior during feeding, play, and distress-eliciting tasks during which infant heart rate is recorded to derive measures of vagal withdrawal; (c) anthropometric measures of mothers and infants; and (d) assays of maternal prenatal blood and infant saliva and urine. A host of demographic and other potential confounds will be considered as potential covariates in structural equation models that include tests of mediation and moderation. Efforts to mitigate the deleterious effects of COVID-19 on study success are detailed.

**Discussion:**

This study has the potential to inform (1) basic science about early life processes casually related to childhood obesity and (2) development of targeted intervention and prevention approaches that consider mother, infant, and family risks and resources.

## Background

Childhood obesity is a critical public health problem, with over 42 million children being overweight or obese worldwide [1]. Further, over half of children today are predicted to be obese by the time they are 35 years old [2]. It is difficult to alter the trajectory of weight gain in an obese child [3], and a host of negative mental and physical health outcomes are associated with the stability of obesity across time [4] . The scientific community has acknowledged the complex nature of obesity, with many factors likely to exert their influence before or during infancy. This developmental complexity is best understood using a framework that includes biological, psychological, and social processes assessed over the course of a child’s early development. However, the pathways to early childhood obesity are largely unspecified because complex models that specify which factors operate as mechanisms (mediators) and which factors affect some individuals and not others (moderators) have yet to be developed and tested [5] .

**Fig. 1:**
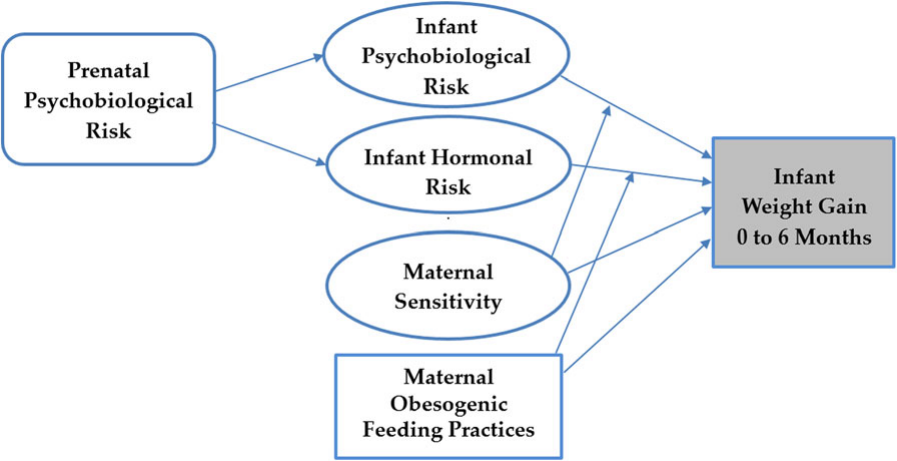
Conceptual Model for Aim 1: Pathways to Infant Weight Gain Birth to 6 Months

**Fig. 2:**
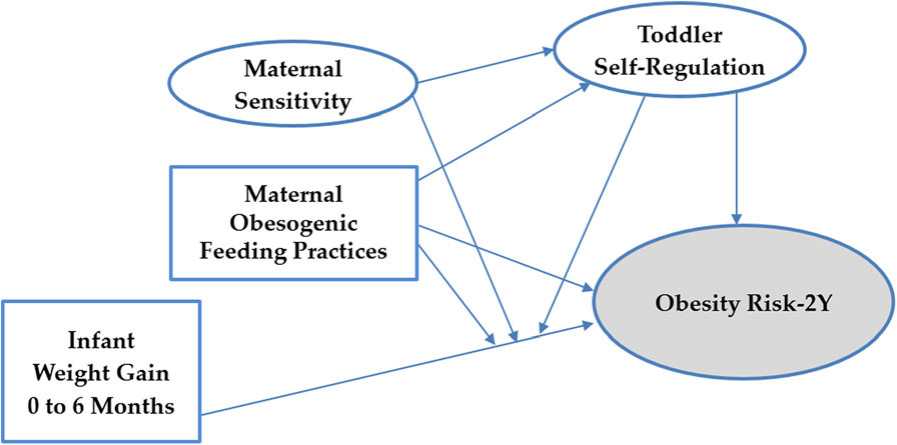
Conceptual Model for Aim 2: Predictors of 2 Year Obesity Risk

In iGrow (Infant Growth and Development Study), we explicitly address several of these gaps via a novel, interdisciplinary, longitudinal, multi-method study to identify childhood obesity foci that will inform future prevention and intervention programs. We take a *biopsychosocial perspective,* focusing on the interplay among psychobiological and hormonal risk prenatally, social processes during early infancy that include maternal sensitivity and feeding practices, and child psychological processes, including child self-regulation. In doing so, we test two overarching aims (Figs. 1 and 2 respectively): (1) to predict infant weight gain from birth to 6 months, with an emphasis on testing interactions between early infant hormonal and psychobiological risk and maternal feeding practices and maternal sensitivity; and (2) to determine whether maternal behaviors and child self-regulation from 14 months through 24 months interact with infants’ early weight gain to predict obesity risk at age 2.

### Primary aim 1: pathways to infant weight gain from birth to 6 months

Our first goal is to identify predictors of infant weight gain, defined as change in weight for length z-score from birth to 6 months of age, because this is a consistent predictor of increased obesity risk later in life [6–11]. Prior research demonstrates that fetal exposure to a host of maternal psychological and biological risk factors such as prenatal stress, elevated maternal weight and related hormones, pregnancy complications, and substance use are linked to children’s subsequent obesity [12–15], but *how* fetal exposure is predictive of later weight outcomes is unclear; given the many factors that affect weight gain, there are likely multiple pathways. We address two mechanisms, or mediational pathways, that might explain the relation between prenatal risk and early weight gain: (1) psychobiological influences that alter infants’ biological and behavioral responses to feeding, and (2) prenatal exposure to a hormonal milieu that may disrupt infants’ normal neural and hormonal regulatory loops.

Infant psychobiological risk, which refers to a cluster of characteristics that represent the infant’s propensity toward hyperactive stress psychophysiology (indexed by elevated HPA-axis activity) and temperamental negativity (indexed by low resting cardiac vagal tone and by irritability and distress to challenge), might serve to mediate the association between prenatal biological risk and greater early weight gain in infancy, via their link with satiety. In prior research, mothers’ prenatal malnutrition, heightened stress, and elevated cortisol were associated with infants’ increased cortisol reactivity, lower basal vagal tone, and heightened irritability [16–23]. In turn, infants who display these psychobiological characteristics are easily aroused, challenging to soothe, and highly reactive to naturally occurring changes and events in their environments. For these infants/caregivers, feeding might serve to provide relief for both, and the associated overfeeding can lead to excessive early infant weight gain [20, 21]. This proposition is consistent with findings of previous studies that linked atypical patterns of cortisol response to stress [22–25], low basal vagal activity [26, 27], and high temperamental fussiness in infancy [20] with eating in the absence of hunger, emotional eating, and elevated BMI.

Infant hormonal risk, defined as elevated levels of leptin and insulin and low levels of adiponectin (hormones related to energy balance and metabolism), may also mediate the association between prenatal biological risk and greater early weight gain. Elevated prenatal risk (i.e., maternal obesity, gestational diabetes) may expose the fetus to disruptive hormone profiles that negatively influence normal development during critical windows. For example, higher maternal leptin concentrations are linked with fetal insulin resistance, [28, 29] and higher infant birth weight, [26, 27, 30, 31] and adiposity [31, 32], but are also negatively associated with early infant growth [28, 33–35]. Likewise, elevated maternal adiponectin has been associated with higher birth weight [36] and greater infant adiposity, [32] but there is a negative association between adiponectin and BMI-for-age in childhood [27] and with obesity and insulin resistance in adults [26, 27]. It may be that an infant’s production of these hormones is more closely predictive of weight outcomes than is fetal exposure. Thus, assessing both and examining them simultaneously in relation to infant weight gain is a critical gap in the existing literature that we are poised to address.

Although we propose that infant psychobiological and hormonal risk predict greater early infant weight gain, we also hypothesize that these associations will not be observed in all infants but will be moderated by caregiving behaviors, with some types of behaviors increasing the risk for obesity and others lowering the risk. We focus on two distinct aspects of maternal behavior: feeding practices and sensitivity. So called “obesogenic feeding practices” refer to a cluster of behaviors that have been associated with increased obesity risk in previous research including formula feeding, introducing solids prior to 4 months, feeding on a schedule, bottle propping, TV watching while feeding, and feeding to soothe [37–42]. In addition to main effects of these practices on infant weight gain, obesogenic feeding practices might also moderate the association between infant hormonal risk and weight gain in the first 6 months. That is, infants who have hormonal risks that predispose them to greater weight gain, coupled with caregiving behaviors that promote overfeeding and/or formula over breastmilk are at greater risk for maladaptive weight outcomes. Some previous research supports this hypothesis by showing a strong link between lower prenatal exposure to leptin and infant weight gain among formula-fed infants compared to breastfed infants [43]. To date, the extent to which infant hormonal risk and obesogenic feeding practices, other than formula feeding, interact to predict infant weight outcomes in early life has not been tested.

Maternal sensitivity, or the promptness and appropriateness of maternal responses to infant signals [44], has been associated with lower risk for overweight and obesity in infancy and later childhood [45–48]. Because these effects might be situational, we focus on sensitive maternal behavior in three contexts: feeding, play, and distress-eliciting tasks. Mothers who are responsive during feeding, read infant hunger and satiety cues accurately and respond appropriately may promote infants’ awareness of and appropriate response to their internal hunger and satiety cues, preventing overeating and greater weight gain [43]. Likewise, sensitivity during play, and even more so during distress-eliciting tasks, prevents and reduces infant distress in the moment [49], making it less likely that mothers will habitually use feeding to soothe [50]. Additionally, sensitive maternal behavior may moderate the extent to which infant psychobiological risk predicts greater infant weight gain. Easily distressed infants tend to elicit more feeding for soothing [40], but sensitive mothers who are skilled at discerning infant cues may be less likely to engage in these behaviors than less sensitive mothers. Thus, maternal sensitivity may reduce the positive association between infant psychobiological risk and early infant weight gain. Prior evidence supports this moderating effect of sensitivity on the link between temperamental fussiness and later weight outcomes [47, 48], but this moderating effect has not been tested with a multi-dimensional measure of infant psychobiological risk that includes physiological, behavioral, and mother-reported indicators.

### Primary aim 2: moderators of the link between infant weight gain and obesity risk at age 2

In the current study, we define obesity risk at age 2 as a composite of high BMI-for-age z scores, adiposity (body fat), and elevated inflammatory markers. This approach is useful because measures of fat mass, regional fat distribution, and inflammatory markers are better indicators of later obesity and related health risks than is BMI alone [51, 52], fat mass and BMI-for-age patterns are distinct among children under age 6 [53], and there has been a strong call for greater attention to body composition and inflammation in infant research [5, 54]. The association between weight gain in infancy and later obesity is well established [6–9, 55–58], but the role of parenting and children’s development of self-regulatory skills in moderating this association remains a significant gap.

As older infants master new eating skills and gain autonomy, the nutrient density of foods and beverages consumed become increasingly important [59, 60]. High intakes of empty calories and low consumption of nutrient-dense foods, such as fruit and vegetables, during infancy has been linked with increased obesity risk [60, 61]. During this period, maternal/parental controlling behaviors (i.e., pressure, restriction, feeding to soothe) also represent obesogenic feeding practices, with links to poor weight outcomes identified in some, though not all studies [42, 62]. We propose that obesogenic feeding practices after 6 months predict obesity risk at the age of 2 and moderate the link between infant weight gain and obesity risk at age 2; children who are already predisposed to weight problems will be at even greater risk for obesity when mothers engage in obesogenic feeding practices [63, 64].

In addition to obesogenic feeding practices, we propose that child self-regulation functions as another key moderator of the link between infant weight gain and obesity risk at age 2. Self-regulation refers to self-initiated control processes that allow a child to manage physiological, attentional, and emotional arousal [65]. Several scholars have suggested that the key mechanism by which maternal sensitivity and feeding practices are linked with children’s later obesity is via their effects on children’s self-regulation [45–47, 66–68] by enhancing a child’s ability to eat according to hunger and satiety cues rather than engage in emotional eating/eating to soothe. Yet, to our knowledge no prior study has directly tested this mediated pathway using a prospective design. Further, the association between early self-regulation and weight outcomes has been tested with preschool and older children [69–72], but not with infants. We propose that higher child self-regulation will be associated with lower obesity risk at age 2, and that children who demonstrate a propensity toward greater infant weight gain, but go on to develop adaptive self-regulation skills, will be buffered from later obesity risk because they are less likely to turn to food for comfort and more likely to respond to their satiety cues.

## Methods/design

iGrow is a prospective, longitudinal, multi-method study following pregnant women and their infants across 5 waves of data collection: 3rd trimester of pregnancy, and 2 months, 6 months, 14 months, and 24 months post-partum. Employed methods include biological assays, physiological assessments, anthropometrics, medical records, dietary intakes, surveys, and direct observations with behavioral coding.

### Setting

iGrow is being conducted in Greensboro, North Carolina, a midsized southeastern city in Guilford County (population 533,670). According to Census data, the racial/ethnic composition of the county is 55% White alone, 34% Black or African American alone, and 11% other/multiple races (8% Hispanic/Latino). Median income for families with children under 18 living in the household was $56,794 and the poverty rate among these families was 20.3% in 2018. Among women aged 25 to 34, 8% had not completed high school/GED and 47% had earned a Bachelor’s degree or higher. In 2015, 29% of 2-to-4-year olds in the county were obese or overweight at their Well Child Visits [73].

### Recruitment and retention

Pregnant women (*N* = 300) and their infants are the participants of this study. Inclusion criteria for mothers are 18 or older, expecting a singleton, fluent in English, and planning to remain in the region for 3 years, to facilitate retention. Infants with birth defects, metabolic disorders, and/or gestational age < 32 weeks are subsequently excluded. Expectant mothers are recruited during their 3rd trimester from (a) childbirth education classes at local hospitals and the Public Health Department, (b) prenatal breastfeeding classes provided by the Special Supplemental Nutrition Program for Women, Infants, and Children (WIC), (c) advertisements in waiting rooms of OB/GYN practices and stores/events targeting expectant parents, (d) via social media (e.g., listservs, Facebook, Twitter), and (e) via paid advertising in regional print and online magazines focused on pregnancy and parenting. The sample generally reflects the racial composition of the surrounding county as described above, except we will strive to oversample Hispanic children (12%, defined as one or both biological parents being Hispanic) given this is a rapidly growing ethnic group in the US at high risk for childhood obesity [74, 75]. Those who meet criteria are invited to participate. One staff member serves as the liaison with participating families to promote rapport and continuity; she is the primary recruiter and schedules all assessments. Childcare and transportation (within a 25-mile radius) are provided for participants as needed. In the event families move from the area, we arrange long distance visits to collect data. If families are unable to participate in data collection lab visits at any time point, they are given the option of completing surveys. Mothers are mailed a congratulation card after the child’s birth. Biannual newsletters and annual mothers’ day cards and infant birthday cards are mailed as retention strategies. Mothers provide information for up to 5 contacts so that we may reach out to in the future should we have difficulty reaching them for follow-up assessments. The incentive for the prenatal visit is $50, and the amount increases at each subsequent visit ($405 total across all 5 waves). Mothers and children receive small gifts with the iGrow logo at each assessment.

### Data collection procedures

#### Prenatal wave

Expectant mothers visit our campus laboratory 6 to 8 weeks prior to their due date. Prior to the visit, they are sent a secure email link to complete questionnaires via Qualtrics. They are asked to fast for 2 h prior to the visit. Participants undergo anthropometric measures (e.g., weight, height, arm circumference), provide a blood sample, and complete a variety of additional questionnaires (i.e., dietary screeners). Blood samples are obtained via venipuncture by a trained research technician using universal precautions. All blood samples are de-identified, processed within 20–30 min, pipetted into small aliquots to limit freeze/thaw cycles and stored at − 80 °C. Participants sign a medical release allowing us to contact OB/GYNs for select information in the event they are unable to provide needed data via the patient portal in the future.

#### Post-birth call

Five days after the due date, the scheduler calls mothers to collect information on the infant (e.g., birthdate, gender, name, birth weight/length), birth (e.g., birth type, complications), to confirm eligibility for continued participation, and to remind mothers of the 2 month visit.

#### 2, 6, 14, and 24 month postpartum waves

The scheduler calls the mother to schedule a home or laboratory visit within +/− 5 days of the infant’s 2-month birthday and + 2 weeks of children’s 6, 14, and 24-month birthdays. Prior to each visit, mothers are sent a secure link to complete questionnaires. Two staff members conduct the visits and each visit lasts 1.5 to 2 h. Mothers complete a brief paper form describing the infant’s feeding, sleep, mood, and health within the last 24 h. With maternal assistance, staff attach a hospital-grade urine bag to the genital area and place cotton balls in the diaper for sample collection. Then, infants are weighed on a calibrated high-precision pediatric scale (Seca, Hamburg, Germany) and the weight of the urine bag and cotton balls are subsequently subtracted. Recumbent length of infants is measured to the nearest 0.1 cm using an infant measuring board (Perspective Enterprises, Portage, MI), 5 skinfolds (i.e., tricep, subscapular, suprailiac, thigh, bicep) are measured using a Lange skinfold caliper, and waist and mid-arm circumference is measured with a Gullick tension tape measure, following standard procedures [76]. Each measure is duplicated for accuracy, and a third measurement is taken if the first two measures disagree by previously established maximum values. At the 24-month visit, the child’s height is measured, using a SECA 214 portable stadiometer with shoes removed. Then, children are fit with pediatric electrodes on their right collarbone, lower left rib, and navel to collect heart rate using the Biolog (UFI, Moro Bay, California) and engage in a resting baseline (10 min sleep sample at 2 months and passive video watching for 2 min at 6, 14, and 24 months) for use in calculating vagal withdrawal scores. Next, mothers and infants engage in a series of video-recorded tasks including: feeding session, 7-min free play, and interactive distress-eliciting tasks. The distress eliciting tasks have been used in prior research and are as follows: Face to Face Still Face [77] at 2 months, a gentle arm restraint and the Face to Face Still Face at 6 months; attractive toy in a locked jar and novel character approach at 14 months; and attractive toy in a locked box and spider approach at 24 months [78, 79]. During the distress tasks, other than the Face to Face Still Face, at 6, 12, and 24 months, mothers are asked to remain uninvolved for the first minute, then to interact with their children as they wish for the remaining 3 min of each task. This procedure allows us to measure child affect and regulation independent of the mother and then to measure maternal sensitivity during distress tasks.

In addition, some procedures are specific to certain waves. *At 2 months only,* mothers log into their patient portal via their OB/GYN’s website to report the results of their oral glucose tolerance test and any diagnoses related to prenatal risk (e.g., gestational diabetes, preeclampsia) and their pre-pregnancy and end-of-pregnancy weight. In the event they are unable to do so, OB/GYNs are contacted to provide the information utilizing the medical release provided prenatally. To assess cortisol reactivity and recovery, infant saliva is collected using Salimetrics SalivaBio’s Infant Swab Method. Data collectors hold the swab in the child’s mouth for up to 2 min, gently moving it around inside the mouth as non-intrusively as possible. Saliva samples are collected upon arrival (baseline 1), 15 min later (baseline 2), and 20 (reactivity) and 40 min (recovery) after the Still Face episode (peak stressor) [80]. Infant heart rate is recorded as described above during a 10-min sleeping/resting period.

*At 14 and 24 months,* infants watch a brief (4.5 min) cartoon on an iPad placed 1.5 ft in front of them while seated in a high chair or on their mothers’ laps to assess the extent to which they attend to and away from the video; this task is video-recorded for subsequent coding. Additionally, mothers complete a modified version of the Food Frequency Questionnaire [81] on which they report their infants’ food intake for the past 7 days, using a supplemental form if the infant was in childcare or in care of other adults who were responsible for feeding during the 7-day period. *At 24 months,* each child will sit in the BODPOD for body composition assessment using a specialized pediatric seat insert validated with children weighing 12 kg or more. Two weeks prior to the 24-month visit, mothers will be mailed Actigraph® accelerometers (Pensacola, FL) with instructions to measure the child’s activity level and sleep for 7 days prior to the visit. Upon arrival at the lab, a staff member will remove the accelerometer from the child.

All biological samples (blood, urine and saliva) will be processed, divided into small aliquots—identified only with date, time, and subject code, and stored at − 80 °C for later analysis.

### Measures

Constructs, measures, sources, and timing of assessment for the primary aims are summarized in Table 1 (primary measures) and Table 2 (covariates). All measures have been used in prior research, have sound psychometric properties, including internal consistency, inter-rater reliability, and predictive and/or convergent validity. Inter-rater reliability for behavioral coding is calculated for 15–20% of observations.

**Table 1:** Measures Related to Primary Aims

Domain	Construct	Srce	Measure	Timing	
				Aim 1	Aim2
Prenatal Psychobiological Risk	Weight Status	LR	Pre-pregnancy BMI	PreN	
		LR	Pregnancy weight gain		
	Pregnancy complications	LR	Pre-eclampsia	PreN	
		LR	Gestational diabetes		
	Insulin Resistance	LR	OGTT results	PreN	
	Hormonal risk	BIO	Leptin, Adiponectin, Insulin	PreN	
	Substance use	MR	Modified WHO ASSIST	PreN	
	Prenatal Stress	MR	CES-D, State-Trait Anxiety Inventory, Stressful Life Events Questionnaire	PreN	
Infant Psychobiological Risk	HPA axis functioning	BIO	Cortisol reactivity-saliva	2 M	
	Cardiac functioning	BIO	Resting vagal tone	2 M	
	Negative emotionality	OBS	Irritability rating	2 M	
		MR	IBQ-VSF	2 M	
Infant Hormonal Risk	Hormone levels	BIO	Leptin, Adiponectin, Insulin	2 M	
Maternal Obesogenic Feeding Practices	Feeding Practices	MR	Infant Feeding Practices Q II	2 + 6 M	14 + 24 M
			Infant Feeding Style Ques.	2 + 6 M	14 + 24 M
			Feeding to Soothe Scale	2 + 6 M	14 + 24 M
			Infant Food Frequency Questionnaire		14 + 24 M
Maternal Sensitivity	Feeding task	OBS	Sensitivity/responsive feeding ratings	2 + 6 M	14 + 24 M
	Play task	OBS	Sensitivity ratings	2 + 6 M	14 + 24 M
	Distressing task	OBS	Sensitivity ratings	2 + 6 M	14 + 24 M
Child Self-Regulation	Physiological	BIO	Vagal withdrawal to stressful tasks		14 + 24 M
	Emotional	OBS MR	Observed affect and regulatory behaviors during stressful tasks IBQ/ECBQ VSF		14 + 24 M
	Attentional	OBS MR	Fixation task ECBQ VSF		14 + 24 M
Infant Weight Gain 0 to 6 Months	Change in WHO Weight-for- length z-score	MR OBS	Weight/recumbent length	6 M – Birth	
Obesity Risk 2Y	BMI-for-age z-score	OBS	Weight/height^a^		24 M
	Adiposity z-score	OBS	Skin folds^a^		24 M
			BODPOD (infant option)		24 M
	Inflammatory markers	BIO	IL-6, TNF-α, CRP^a^		24 M

*OBS* observed, *BIO* biological measure, *LR* official lab reports/Patient Portal, *MR* mother reported, *PreN* prenatal, *M* months; + = composite across time, − is a change score

^a^To be assessed at each time point for secondary analyses; timepoint related to primary aims is noted in table

**Table 2:** Covariates

Domain	Construct	Srce	Measure	Timing	
				Aim 1	Aim2
Demographics	Mother/infant race/ethnicity Family income^a^ Food insecurity Maternal education Maternal age Marital status^a^ Father’s presence in the home^a^ Infant birth order Onset of prenatal care Infant age at assessment	MR	Demographic questionnaire	PreN/6 M^1^	PreN/24 M^1^
	Infant sex Gestational age Delivery mode Birth complications Birthweight/length	MR	Post-birth phone call	1wk	1wk
	Childcare onset, frequency, type	MR	Demographic questionnaire	2 + 6 M	14 + 24 M
Neighborhood characteristics	Neighborhood conditions	MR	Neighborhood scales	PreN	24 M
Infant competing correlates of weight outcomes	Infant sleep	MR OBS	Brief Infant Sleep Questionnaire Actigraph	2 + 6 M	14 + 24 M 24 M
	Infant activity level	MR OBS	IBQ-R VSF Actigraph	6 M	14 + 24 M 24 M
	Infant opportunity for movement	MR	Physical Activities and Pursuits	6 M	14 + 24 M
	Infant screen time	MR	Common Sense Census	6 M	14 + 24 M
	Infant energy intake	MR	Infant Food Frequency Questionnaires		14 + 24 M
	Infant concurrent hormonal risk	BIO	Leptin, Adiponectin, Insulin	6 M	24 M
Maternal competing correlates of weight outcomes	Concurrent depressive symptoms (unmeasured parenting effects)	MR	CES-D	2 + 6 M	14 + 24 M
	Concurrent BMI (genetics)	OBS	Weight/height	6 M	24 M
Potential COVID related controls	COVID-related stressors Assessment date relative to COVID events	MR OBS	Additions to Stressful Life Events	PreN, 2 M	14 + 24 M

*OBS* observed, *BIO* biological measure, *MR* mother reported, *Srce* Source, *PreN* prenatal, *M* months; + = composite across time

^a^Items repeated given they change over time

#### Prenatal psychobiological risk

*Maternal weight status* is indexed by maternal pre-pregnancy BMI and pregnancy weight gain derived from lab reports or self-report (2 risk factors). *Pregnancy complications* is indexed by diagnoses of gestational diabetes and/or pre-eclampsia (2 risk factors) and *insulin resistance* is indexed via OGTT results (1 risk factor) each of which will be obtained from lab reports. *Hormonal risk* is indexed via assessment of blood levels of insulin (Sigma, St. Louis, MO), leptin, and adiponectin (both from R & D Systems, Minneapolis, MN), using ELISA technology (3 risk factors). Mothers report on *tobacco, alcohol, and cannabis use* via a modified version of the Alcohol, Smoking and Substance Involvement Screening Test (ASSIST) [82] on which they report use by trimester (1 risk factor). Mothers’ *prenatal stress* is indexed by self-reports on the CES-D (depression) [83], the State-Trait Anxiety Inventory [84], and the Stressful Life Events Questionnaire [85] (3 risk factors). A proportional composite risk index will be created using a sum of risk factors in which dichotomous risks are coded as 0 = no risk and 1 = risk, and continuous risks (i.e., depressive symptoms, anxiety, stressful events, substance use) are converted into proportions of the possible total score and thus range from 0 to 1 while preserving maximum variability [86, 87]. Total prenatal risk scores potentially range from 0 to 12.

#### Infant psychobiological risk

*HPA-axis functioning* is indexed by cortisol reactivity and recovery following the Face to Face Still Face during the 2-month visit. Assays will be conducted using Salimetrics Salivary Cortisol ELISA Kits. Samples will be run in duplicate with greater than 20% variation between duplicates as the criterion for repeat analysis. *Cardiac functioning* is operationalized as a 5-min quiet sleep sample of respiratory sinus arrhythmia (RSA) derived from heart rate data using Porges’ method [88]. *Infant negative emotionality* is assessed using two measures at 2 months. Infant irritability is rated on a 7-point scale during the free play task and each phase of the Face to Face Still Face; scores will be averaged across tasks to yield a single measure of irritability [89]. And at 2 months, mothers complete the negative affectivity subscale of the Infant Behavior Questionnaire-Revised Very Short Form (IBQ-R VSF) [90].

#### Infant hormonal risk

*Hormone levels* are derived from urine samples; the 2-month sample serves as our measure of infant hormonal risk to test Aim 1. Urine osmolality (solute level in urine) will be measured in duplicate using the freezing point depression method (Model 3320, Advanced Instruments, Norwood, MA) to control for infant hydration. Assessment of all infant biomarkers will be done using commercially available ELISAs (leptin; BioVision [Milpitas, CA], adiponectin; Quidel [San Diego, CA], insulin; Sigma [St. Louis, MO]). All samples from an individual will be assayed in a single kit to minimize the effects of inter-assay variability.

#### Maternal obesogenic feeding practices

*Feeding type (*breastmilk, formula, mixed*), feeding mode (*percent of feeds from bottle)*, duration of exclusive breastfeeding, timing of solid food introduction,* and *maternal provision of* sugar-sweetened beverages, sweets, high fat foods, fruit, and vegetables are assessed via maternal report on an adaptation of the Infant Feeding Practices Questionnaire II [91]. *Uninvolved feeding, pressure feeding, and feeding to soothe* are assessed via maternal report on the Infant Feeding Style Questionnaire [92] and the Feeding to Soothe Scale [40]. A proportional composite risk index will be created using a sum of obesogenic practices. Dichotomous risks are coded as 0 = no risk, 1 = risk (e.g., introducing solids prior to 4 months). Continuous (e.g., feeding to soothe, pressure feeding) and ordinal risks (e.g., exclusive breastmilk [0], mixed [1], formula only [2]) will be converted into proportions of the possible total score and range from 0 to 1. Obesogenic feeding practices potentially range from 0 to 6 at 2 and 6 months and 0 to 8 at 14 and 24 months.

#### Maternal sensitivity

Maternal behavior during the free play and distress eliciting tasks is rated on the following dimensions using a 7-point scale: sensitivity to distress, sensitivity to non-distress, intrusiveness, detachment, positive regard and negative regard [93]. The same coding system was adapted for the feeding task by modifying descriptors/anchors for the above dimensions and by adding the dimension sensitivity to fullness cues based on other feeding assessments such as the Nursing Child Assessment Feeding Scale [94, 95].

#### Child self-regulation

*Emotion regulation* is measured by direct observation of expressed affect (e.g., latency to distress, peak distress) and regulation behaviors (e.g., self-soothing, bids for assistance, problem solving) during the distress eliciting tasks at 14 and 24 months [79, 96]. Additionally, mothers report on child negative affect and soothability/ falling reactivity/rate of recovery using the Early Childhood Behavior Questionnaire Short Form (ECBQ SF) at 24 months [97]. *Physiological regulation* is measured as vagal withdrawal, or RSA change, from the passive video baseline to the distress-eliciting tasks at 14 and 24 months. *Attentional control* is measured by direct observation of attention duration and shifting during the fixation video task [98] and maternal report of attentional focusing on the ECBQ SF.

#### Infant weight gain

Infant weight gain is operationalized as *change in weight-for-length z-scores from birth to 6 months* using WHO growth standards as recommended by the CDC for children under age 2 [99, 100].

#### Obesity risk at age 2

Obesity risk at age 2 is measured by age and sex-specific *BMI-for-age* z-scores derived from gender specific WHO growth standards [99, 100], gender- and age-specific *adiposity z-scores* from the *BODPOD*, and from *inflammatory markers* (as described for hormonal risk), using commercially available ELISAs (IL-6; Thermo [Waltham, MA], TNF-α; Abcam [Cambridge, MA] and hs CRP; ALPCO [Salem, NH]). All three measures will be used to construct a latent variable in analyses.

#### Potential covariates

Mother/infant *race, family income, maternal education and age, marital status, fathers’ presence in the home, infants’ birth order,* and *onset of prenatal care* are reported by mothers prenatally on a demographic questionnaire and information is updated as needed at each assessment point. *Infant biological sex, birth weight, gestational age, delivery mode, and birth complications* are reported by mothers during the post-birth phone call and used to determine continued eligibility to participate or as possible covariates. Items about *childcare onset, type and frequency* are included on the demographic questionnaire 2, 6, 14, and 24 months. Mothers report on *neighborhood conditions* (including walking environment, availability of healthy foods, safety*)* prenatally and at 24 months [101]. At 2, 6, 12, and 24 months, mothers report on *infant sleep* using the Brief Infant Sleep Questionnaire [102] and infant activity level using the IBQR VSF and then ECBQ SF. *Infant sleep* and *physical activity* are directly measured at 24 months using a 24-h hip-worn accelerometer protocol with vector magnitude counts [103, 104] as well as standard cut points for sleep and physical activity [105–108]. In pediatric populations, a minimum wear time of 8 h across 3 days is needed for accurate assessment of physical activity [109], while 5 days is required for accurate sleep assessment [106]. Wear time will be examined as a potential covariate as well. The child’s *average daily energy intake (kilocalories)* at 14 and 24 months will be calculated based on maternal reports on the Food Frequency Questionnaire [81]. Mothers report infants’ *screen time* [110], and opportunities for movement [111] at 6, 12, and 24 months. *Child hormonal risk at 24 months* is measured as described above. *Maternal BMI* at 6 months and 2 years, derived from height and weight as assessed during those visits, and *depressive symptoms* reported on the CES-D [83] at 6 months and 2 years will serve as covariates (prenatal measures of each are primary variables).

### Statistical plan

#### Overview

After examining the psychometric characteristics of each measure, executing any needed transformations, and screening potential covariates for inclusion, the analyses will be conducted using structural equation modeling (SEM). The models will include latent constructs (ovals in Figs. 1 and 2) and manifest constructs (boxes in Figs. 1 and 2). The measurement models for the latent constructs will be examined, followed by estimations of structural models to test specific hypotheses. In addition to reducing estimation bias by minimizing measurement error, a major strength of SEM is that it can accommodate the conjoint testing of mediation and moderation. Missing data will be addressed using full information maximum likelihood (FIML) estimation methods, which produce less biased estimates than other methods [112].

#### Examining aim 1

Our first aim is to test a model predicting infant weight gain from birth through 6 months from prenatal psychobiological risk, infant psychobiological and hormonal risk, maternal sensitivity, and obesogenic feeding practices (see Fig. 1). Infant weight gain will be a continuous change score that reflects changes in weight-for-length z-scores from birth to 6 months (manifest variable). Prenatal psychobiological risk will be a manifest variable (described above). Infant psychobiological risk and infant hormonal risk at 2 months are expected to be modeled as formative latent variables. In formative latent constructs (also called causal latent constructs), measurement variables might not be highly correlated but are needed to adequately represent the latent index [113]. Averaging 2- and 6-month data to enhance reliability [114, 115], maternal sensitivity demonstrated during three tasks is expected to be modeled by creating a reflective latent construct. In reflective latent constructs the measurement indicators are expected to converge, as denoted by moderate-to-high factor loadings. The manifest index of obesogenic feeding practices also will be averaged across 2- and 6-month data. These SEM analyses will control for several potential selection and confounding variables, including race, socioeconomic status, access to parks, family composition, infant birth mode, birth weight, sex, and infant sleep (Fig. 1).

The significance of the two hypothesized mediating pathways will be estimated by a bootstrapping approach, because this method makes no assumptions about sampling distribution and because it has higher statistical power than other estimation approaches [116]. The significance of the hypothesized two moderating effects will be tested using latent interaction terms [117].

An initial sample size of 300 will result in a sample size of 250 conservatively estimating 17% postnatal attrition yielding adequate statistical power to test this model and these hypotheses. We need a sample size of at least 250 to distinguish between a good fitting model (RMSEA = .050) and an adequate fitting model (RMSEA = .065) at a power level of .80 [118]. We also completed a longitudinal data simulation of the models for testing Aim 1 and 2. The model was adequately powered to find a significant statistical interaction effect (β = .16, *p* = .018).

#### Examining aim 2

The purpose of Aim 2 is to identify maternal and infant predictors of obesity risk at age 2 (Fig. 2). Risk for obesity at age 2 will be a reflective, continuous latent construct with three indicators each assessed at 2 years: WHO BMI-for-age z-score, adiposity, and inflammatory markers. Aggregating data from 12- and 24-month assessments to enhance reliability, the predictors of toddler self-regulation and maternal sensitivity will be created as reflective latent constructs. The manifest obesogenic feeding practices variable also will be created using data collected at 14 and 24 months. The simulation of the model in Aim 2 suggested that at least 150 degrees of freedom will be needed. The SEM analyses will be adequately powered to find an adequate model fit (as it was for the Aim 1 simulation), and a statically significant interaction (β = .20, *p* = .002).

### COVID-19 related variations and efforts to address

The COVID-19 pandemic struck in spring 2020, leading to the suspension of all in-person data collection with human participants in mid-March. Following extensive vetting of a risk-mitigation protocol that included staff wearing full personal protective equipment, screening mothers, infants, and personnel for COVID-19 symptoms and exposures (e.g., enhanced cleaning, use of Personal Protective Equipment), postnatal data collection for previously enrolled participants resumed in mid-July 2020 and the needed assessments for catch-up were conducted by October, 2020. Given continued high rates of community spread and pregnant women’s higher risk status for COVID-19 complications, recruitment and prenatal data collection remain suspended at the time of the current manuscript submission. As a result of this unprecedented public health crisis, several steps were taken or are planned to ensure completion of this project and to minimize missing data.

First, we now consider this a 2-cohort design in which cohort 1 includes women recruited prior to COVID-19 related suspension of research (*n* = 175) and cohort 2 will consist of women enrolled post-suspension of activities. We anticipate recruiting a minimum of 130 additional women to achieve the initial goal of 300 and to replace some participants who might attrite due to COVID-19. Second, we added 4 items to our measure of life stressors for use as possible covariates: tested positive for or had reason to believe you had COVID-19/coronavirus; a family member of close friend tested positive for or had reason to believe they had COVID-19; had to care for/work with individual who tested positive for or suspected they had COVID-19; COVID-19/Coronavirus altered your daily routine. Participants indicated whether each occurred, and then rate the extent to which it affected them. We have also recorded the dates of significant local events related to COVID-19 (e.g., local public schools closed, Governor mandated stay at home order, etc.) and will calculate the difference between assessment dates and these dates to determine if date of visit relative to COVID-19 events is a needed covariate. Third, we implemented a host of measures to minimize missing/late data during this period. For instance, we (a) continued to administer online surveys at the planned time, and converted any questionnaires typically completed during home or lab visits for online administration, (b) arranged for contactless pick-up of urine samples by leaving supplies and instructions in an agreed upon location and then retrieving samples from that location, and (c) secured medical releases from participants to obtain infant height and weight data from well-child visits. Finally, when allowed to resume data collection we extended our windows for data collection as follows: 2-month visits may occur from 2 to 4.5 months; 6-month visits may occur from 6 to 9 months, and 14 month visits may occur from 14 to 18 months. 24-month visits are projected to begin on time in spring 2021. For infants who timed out of 2-month in person data collection because of COVID-related halt to in-person data collection, cortisol samples are being collected at 6 months given the same stressor task (Face to Face Still Face) is administered at both. Although less than ideal, these measures will greatly reduce the amount of missing data due to COVID-19 and we intend to control for infant age at assessment as needed. The impact of COVID-19 on ongoing research is unprecedented and the total impact on this study remains unknown. Importantly, strong plans are in place to minimize missing data due to COVID and to statistically control for variability based on cohort, timing, and personal experiences with COVID. This will be noted as a limitation in future manuscripts.

## Discussion

The iGrow Study will make several important conceptual, methodological, and translational advances in understanding the complex pathways to infant early weight gain and childhood obesity. First, our approach explicitly addresses several understudied gaps in the understanding of a developmental process that is multifactorial and likely consists of several child and social-environmental factors that interact across time in complex ways. Second, our novel biopsychosocial model of childhood obesity development begins in the prenatal period and is sensitive to the multiple complex pathways to obesity during the developmental period of infancy, when patterns of weight gain emerge and self-regulatory behaviors are becoming important to infant functioning across many domains. Third, we include sophisticated biomarkers for obesity, which may help to explicate the links between obesity risk and other related health conditions, an approach that is highly novel in this age group. Fourth, the study will make important empirical advances by incorporating complex biological and behavioral measures that have rarely been studied during infancy. This approach is invaluable because emerging data suggest that the biological and behavioral processes involved in childhood obesity begin to operate quite early in development. The measurement and analytical approach will use multiple methods across several time points to examine predictors of infant weight gain and obesity risk. Finally, the study has clear implications for intervention and prevention in families with infants and very young children. Although prior work has focused on several of the individual factors that we address in the current study, no research to date has addressed the ways in which these factors may moderate and mediate one another. This is a key distinction between the current study and prior work in this area. Without specifying how these factors work in the context of one another, it is difficult to identify specific points of entry and appropriate targets for prevention and intervention. Indeed, this was the conclusion of the working paper on childhood obesity [5] and our study is uniquely poised to make such a contribution.

## Data Availability

Data sharing is not applicable because this paper describes the protocol for an on-going study.

## Abbreviations

ASSIST: Alcohol, Smoking and Substance Involvement Screening Test
BIO: Biological measure
BMI: Body Mass Index
CDC: Centers for Disease Control
CES-D: Center for Epidemiological Studies Depression Scalel COVID, Coronavirus Disease
CRP: C-reactive hormone
ECBQ VSF: Early Childhood Behavior Questionnaire Very Short Form
FIML: Full information maximum likelihood
HPA: Hypothalamic pituitary adrenal
IBQR VSF: Infant Behavior Questionnaire Revised Very Short Form
IL-6: Interleukin 6
M: Months
MR: Mother-reported
OB/GYN: Obstetrician/gynecologist
OBS: Observed
OGTT: Oral Glucose Tolerance Test
PreN: Prenatal
RMSEA: Root mean square error approximation
RSA: Respiratory sinus arrhythmia
SEM: Structural equation modeling
SRCE: Source
TNF: Tumor necrosis factor
WHO: World Health Organization
